# Are Higher Medical Benefits Associated with Fertility Intentions?—A Survey Study from the Yangtze River Delta Region

**DOI:** 10.3390/healthcare14152363

**Published:** 2026-08-03

**Authors:** Guang Yang, Xiao Yang, Lufa Zhang

**Affiliations:** 1 School of Public Administration, Shandong Normal University, Jinan 250014, China; yangguangyyds@sdnu.edu.cn; 2 School of Labor Relations, Shandong Management University, Jinan 250357, China; 3 School of International and Public Affairs, Shanghai Jiao Tong University, Shanghai 200030, China

**Keywords:** medical insurance, medical services, fertility intentions, total fertility rate, citizens’ compliance, China, Yangtze River Delta region

## Abstract

**Objectives:** The aging population problem caused by low fertility intentions has already emerged worldwide and is expected to become more severe in the future. Despite the gradual implementation of welfare policies, there has been no substantial improvement in individuals’ intentions to have more children. It has therefore become increasingly important to examine the factors that correlate with individuals’ intentions in relation to fertility policy. **Methods:** The data used in this study were drawn from the 2023 Health Life Satisfaction Survey of the Yangtze River Delta Phase II conducted by Shanghai Jiao Tong University. The primary analysis employed binary logistic regression to examine the association between medical insurance type and fertility intentions, with healthcare accessibility operationalized as a moderating variable. Gender and parity-stratified analyses were conducted as pre-specified exploratory secondary analyses to examine potential heterogeneity in these associations. **Results:** A total of 8789 citizens were included in the analysis, of whom 18.42 percent reported fertility intentions. Individuals enrolled in the Basic Medical Insurance System for Urban Employees exhibited significantly higher odds of having fertility intentions compared with those enrolled in the Basic Medical Insurance System for Urban and Rural Residents (OR = 1.2154, 95% CI = 1.0626–1.3901, *p* < 0.001). Moreover, individuals covered by the Basic Medical Insurance System for Urban Employees and residing near secondary hospitals showed significantly higher odds of fertility intentions (OR = 1.6727, 95% CI = 1.1273–2.4821, *p* < 0.05) than those covered by the Basic Medical Insurance System for Urban and Rural Residents and living near primary hospitals. **Conclusions:** Medical insurance benefit levels, as well as the quality and accessibility of healthcare resources, are positively associated with individuals’ fertility intentions, suggesting that welfare design and service delivery may jointly shape the institutional context for reproductive decision-making.

## 1. Introduction

The phenomenon of declining fertility rates has become a significant global issue, impacting economic growth, social stability, and the sustainability of national welfare systems. According to the United Nations (2022) [1], the global Total Fertility Rate (TFR) has decreased from 4.5 births per woman in 1970 to 2.4 in 2020, with many countries falling below the replacement level of 2.1 births per woman. The implications of low fertility rates are wide-ranging and can profoundly affect various aspects of societal development. Economically, declining birth rates can result in a diminishing labor force, potentially impeding productivity and economic growth [2]. Additionally, a smaller working-age population may struggle to support an increasing number of retirees, placing significant strain on social security and pension systems [3]. This demographic imbalance can also exacerbate intergenerational inequalities and intensify fiscal pressures on governments [4]. Beyond economic implications, low fertility rates can also disrupt established social norms and structures, particularly in societies where traditional values emphasize familial ties and intergenerational support [5]. Furthermore, the resulting age imbalances can exacerbate existing gender disparities, as women may face increased caregiving responsibilities for aging relatives, potentially hindering their participation in the labor force and perpetuating gender inequalities [6,7].

In China, the issue of fertility intentions and behavior has been shaped by a complex interplay of historical, cultural, and socioeconomic factors, as well as evolving government policies and initiatives. In the early years following the establishment of the People’s Republic of China in 1949, the government actively promoted a pro-natalist stance, encouraging higher birth rates to support the country’s economic development and nation-building efforts [8,9,10]. This period was characterized by policies and campaigns that celebrated large families and provided incentives for childbearing, such as maternity benefits and preferential access to housing and employment [11,12]. However, by the late 1970s, concerns over rapid population growth and its potential strain on resources and economic development led to a dramatic shift in policy direction. The introduction of the “One-Child Policy” in 1979 marked a concerted effort by the government to curb fertility rates through a combination of strict birth quotas and punitive measures against non-compliance.

While the One-Child Policy was effective in reducing fertility rates, it also gave rise to a range of unintended consequences. With a rapidly aging population and a shrinking labor force, the country faced significant challenges in maintaining its competitiveness and ensuring the financial viability of its pension and healthcare systems [13]. In response to these challenges, the government gradually relaxed the policy, culminating in its replacement with a “Two-Child Policy” in 2015 and further liberalization in 2021 to allow for three children per couple. These policies include financial incentives, such as child allowances and tax benefits, as well as measures to improve work–life balance and support for families, such as paid parental leave and subsidized childcare. Despite these policy shifts, China’s fertility rate has remained stubbornly low. Since 2022, China’s natural rate of fertility has shown negative growth [14].

Despite extensive research on the determinants of fertility rates, including macroeconomic institutional factors and micro-level social capital factors, relatively few studies have examined fertility intentions through the lens of institutional welfare arrangements and their interactions with healthcare accessibility. While China’s pronatalist policy shift since 2015 provides a clear policy context within which individual reproductive decisions are made, the mechanisms through which institutional factors shape fertility intentions remain underexplored. The present study draws on citizen compliance theory and the Theory of Planned Behavior as orienting frameworks to examine the institutional correlates of fertility intentions among urban residents in the Yangtze River Delta.

Against this backdrop, the present study addresses the following research question: Are higher medical insurance benefit levels associated with greater fertility intentions among urban residents in the Yangtze River Delta, and does healthcare accessibility moderate this association? Drawing on citizen compliance theory and the Theory of Planned Behavior as institutional frameworks, this study advances three hypotheses. First, individuals covered by the more generous Basic Medical Insurance System for Urban Employees (BMISUE) are expected to report higher fertility intentions than those covered by the Basic Medical Insurance System for Urban and Rural Residents (BMISURR). Second, individuals with access to higher-tier medical facilities are expected to report higher fertility intentions, independent of insurance type. Third, the association between insurance benefit levels and fertility intentions is expected to be stronger among individuals with better healthcare accessibility, suggesting a moderating role of service delivery conditions.

This study makes two contributions to the existing literature. Theoretically, it extends the citizen compliance framework to the domain of reproductive intentions, theorizing fertility intentions as policy-relevant behavioral responses shaped by institutional arrangements rather than purely private preferences. Empirically, it provides evidence from China’s Yangtze River Delta—one of the country’s most economically developed regions—on the joint role of welfare design and healthcare accessibility in shaping fertility-related decisions, an area that has received limited systematic attention in prior research.

## 2. Literature Review

### 2.1. Policy Compliance and Institutional Trust

The existing literature has identified several key determinants that influence individuals’ willingness to comply with government policies and directives. One prominent factor is the perceived fairness and legitimacy of the policy-making process and the authorities responsible for implementing and enforcing these policies [15,16,17,18]. When individuals perceive the policy-making process as transparent, inclusive, and representative of their interests, they are more likely to view the resulting policies as legitimate and worthy of compliance [19,20]. Another crucial factor shaping policy compliance is the level of trust in government institutions and their ability to effectively implement and enforce policies [21,22,23]. Individuals who have greater confidence in the competence and integrity of government agencies are more likely to comply with their policies, as they perceive these institutions as capable of delivering on their promises and upholding the rule of law [24,25]. Moreover, the perceived quality and effectiveness of public services and the degree to which they meet citizens’ needs and expectations can significantly influence policy compliance [26,27,28]. When individuals are satisfied with the quality and accessibility of public services, they are more likely to view government policies and initiatives as beneficial and worthy of compliance [29,30,31].

In this study, “compliance with fertility policy” refers specifically to expressed intentions to have a child or additional children, interpreted in the context of China’s pronatalist policy shift since 2015, which has encouraged families to have two or three children. We acknowledge that fertility intentions do not fully capture policy compliance in a strict behavioral or legal sense; rather, they represent citizens’ stated reproductive aspirations, which may reflect—among other things—their willingness to act in alignment with pronatalist policy objectives. Throughout the manuscript, we use “fertility intentions in the context of pronatalist policy” and “compliance-oriented fertility intentions” interchangeably, while recognizing this approximation.

### 2.2. Determinants of Fertility Intentions: Individual and Structural Perspectives

In the context of addressing declining fertility rates, understanding the factors that correlate with individuals’ intentions with fertility intentions and related government policies is crucial. Fertility intentions and behaviors are influenced by a complex interplay of individual, socioeconomic, cultural, and institutional factors [32,33,34]. Compliance with policy objectives is a multifaceted phenomenon; a range of previous studies found that it was influenced by a combination of both macro and micro factors [18]. On the micro level, the demographic and socioeconomic characteristics, such as age, education, employment status, income, and marital status, have been consistently identified as significant predictors of fertility intentions and behaviors [35,36]. For instance, studies have shown that higher levels of education and income are often associated with lower fertility rates, particularly in developed countries [37,38,39]. Additionally, women’s labor force participation and career aspirations have been linked to delayed childbearing and lower fertility rates [40,41]. Sociocultural norms and values also play a crucial role in shaping fertility intentions and behaviors [42,43]. These include gender role attitudes, family values, and social norms surrounding childbearing and childrearing. In societies with more traditional gender role attitudes and strong family-oriented values, individuals may be more inclined to have larger families [44,45,46]. Conversely, in societies with more egalitarian gender role attitudes and individualistic values, fertility rates tend to be lower [47,48].

At the macro level, including government policies, welfare state provisions, and the availability and quality of public services, can significantly influence fertility intentions and behaviors [49,50,51]. Policies that support work–life balance, such as parental leave, childcare subsidies, and flexible work arrangements, have been shown to positively impact fertility rates [52,53]. Additionally, the availability and affordability of healthcare services, including prenatal and postnatal care, can shape individuals’ perceptions of the costs and benefits associated with childbearing [54,55].

One of the key institutional factors widely recognized as influencing fertility intentions and behavior is the availability and quality of healthcare services, including medical insurance coverage and associated benefits. Extensive research has explored the impact of medical insurance and healthcare service quality on fertility intentions and behavior, both in China and globally. Medical insurance coverage plays a pivotal role in ensuring access to healthcare services and mitigating the financial burden associated with medical expenses, including those related to childbearing and child-rearing [56,57]. Comprehensive medical insurance coverage can reduce the perceived costs of childbearing, potentially encouraging individuals and couples to have more children [58,59].

### 2.3. Health Insurance, Healthcare Access, and Fertility in China

**Figure 1:**
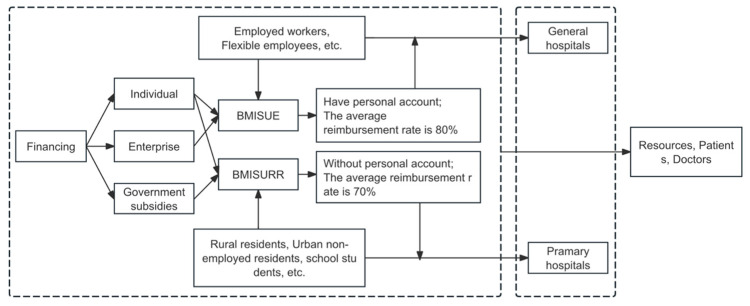
The Current Healthcare Security System in China.

In China, the government has implemented various medical insurance schemes to improve healthcare access and affordability for its citizens. The Basic Medical Insurance System for Urban Employees (BMISUE) and the Basic Medical Insurance System for Urban and Rural Residents (BMISURR) differ substantially in their financing mechanisms and benefit generosity. BMISUE is financed through mandatory contributions from both employers and employees, resulting in higher reimbursement rates, broader service coverage, and more comprehensive maternity-related benefits. In contrast, BMISURR relies primarily on individual contributions and government subsidies, with comparatively lower reimbursement ceilings and more limited benefit packages [60,61,62]. These institutional differences imply unequal levels of financial protection and healthcare security, which may shape individuals’ perceptions of the costs and risks associated with childbearing. Figure 1 shows the current healthcare security system in China.

Several studies have explored the impact of medical insurance coverage on fertility intentions and behavior in China. For instance, Zhang and Huang (2021) found that women enrolled in the social medical insurance programs had higher fertility intentions compared to those without medical insurance coverage [63]. Several studies have examined the impact of the extent of health insurance coverage on fertility intentions. However, data from China National Health Insurance Administration (CNHIA) in 2023 showed that the number of people covered by China’s social medical insurances has reached 1.36 billion, with the participation rate remaining stable at over 95% for four consecutive years [64]. No research has yet explored whether the type of medical insurance, differentiated by levels of benefits, affects the willingness to comply with fertility policies. In light of existing research findings, this paper proposes the following hypothesis.

**Hypothesis** **1.** *Compared to individuals insured under BMISURR, those covered by the more benefit-generous BMISUE are hypothesized to report higher fertility intentions in the context of China’s pronatalist policy environment.*

### 2.4. Healthcare Inequality and the Three-Tier Hospital System

Drawing on the logic of the Theory of Planned Behavior [65], this study treats medical insurance benefit levels as institutional proxies for attitudinal evaluation of childbearing, and healthcare accessibility as a proxy for perceived behavioral control. We note that this application is institutional rather than psychological: key TPB constructs are inferred from structural variables rather than directly measured through individual-level survey items. This approach has precedent in population research that adapts individual-level behavioral theories to macro-level institutional analysis [66,67]. Furthermore, social exchange theory [68] suggests that individuals engage in social exchanges based on the perceived costs and benefits of their actions. When citizens perceive the benefits of government policies and public services as favorable, they may be more likely to reciprocate by complying with policy objectives, such as those related to fertility intentions [69]. Conversely, if individuals perceive public services as inadequate or unsatisfactory, they may be less inclined to comply with policy goals, as the perceived costs of compliance outweigh the perceived benefits [70].

Healthcare accessibility, service availability, and satisfaction with these services are crucial factors influencing compliance with fertility policies. Accessibility, encompassing aspects such as the distribution of facilities, quality of services, availability of medical personnel, and equipment, directly shapes public trust in the healthcare system [71,72]. High accessibility tends to foster trust and encourage adherence to fertility policies, whereas low accessibility may breed distrust and non-compliance. Similarly, satisfaction with healthcare services, including quality of care, professional competence of medical staff, and costs, influences public perceptions of the healthcare system, impacting compliance with fertility-related policies. High satisfaction levels can increase trust and willingness to comply, while low satisfaction may diminish trust and lead to non-compliance [73]. When individuals perceive medical services as inadequate or unsatisfactory, concerns about potential burdens, compromised access, or suboptimal care may discourage them from having additional children, reducing their compliance with policies aimed at promoting higher birth rates.

The Chinese healthcare system is structured around a three-tier hospital network, comprising primary, secondary, and tertiary facilities. At the grassroots level, primary institutions such as village clinics and community health centers provide basic medical services and health education to local populations. Secondary hospitals at the county and district level offer more specialized outpatient and inpatient care, serving as regional referral centers [74]. Tertiary hospitals, often affiliated with universities, are concentrated in major cities and provincial capitals, functioning as advanced referral hubs with cutting-edge diagnostic and treatment capabilities [75]. This tiered system, however, faces challenges in ensuring equitable resource allocation. The distribution of high-quality medical resources is skewed towards more developed urban areas, where tertiary hospitals are predominantly located [74]. This uneven distribution may have implications for public adherence to family planning policies, as individuals’ perceptions of healthcare access and quality can shape their fertility decisions [76,77]. Further research is needed to elucidate the complex interplay between China’s three-tier hospital system and its potential effects on citizens’ compliance with fertility policy.

**Hypothesis** **2.** *Individuals with proximity to higher-tier medical facilities are expected to report higher fertility intentions, independent of insurance type.*

**Hypothesis** **3.** *The positive association between insurance benefit level and fertility intentions is expected to be stronger among individuals with access to higher-tier medical facilities, suggesting a moderating role of healthcare accessibility.*

### 2.5. Conceptual Framework

In the present study, the Theory of Planned Behavior serves as an orienting framework rather than a fully decomposed individual-level psychological model. Inspired by TPB’s core constructs, the analytical framework maps institutional variables onto attitudinal and behavioral-control dimensions at the macro level, without claiming to operationalize TPB in its original psychological form. Specifically, medical insurance benefit levels are theorized to shape individuals’ attitudes toward childbearing by altering perceived financial risks and welfare support associated with fertility. Healthcare accessibility and the hierarchical level of nearby medical institutions are conceptualized as proxies for perceived behavioral control, as they reflect individuals’ assessments of their capacity to manage pregnancy, childbirth, and childrearing within the existing healthcare system. Although subjective norms are not directly measured, they are partially embedded in the broader policy environment and institutional context that signal state expectations regarding fertility behavior.

This study advances a compliance-oriented perspective on fertility intentions by theorizing them as policy-relevant behavioral intentions shaped by institutional arrangements rather than purely private demographic preferences. Anchored in citizen compliance theory, the framework posits that differentiated medical insurance benefit levels constitute a salient institutional signal through which the state structures citizens’ evaluations of policy fairness and reciprocity, thereby influencing their willingness to comply with fertility policy objectives. Drawing on the Theory of Planned Behavior, insurance generosity is expected to shape compliance-oriented fertility intentions by conditioning both attitudinal evaluations of childbearing and perceived behavioral control under policy constraints. Crucially, the framework specifies healthcare accessibility and quality—operationalized as distance to hierarchical levels of medical institutions—as a moderating condition rather than a mediating mechanism: while insurance benefits define formal entitlements, spatial access to healthcare determines the practical credibility of those entitlements. Unequal access to higher-level medical facilities may thus attenuate the extent to which insurance generosity translates into compliance intentions, whereas better accessibility strengthens this linkage. By explicitly modeling healthcare accessibility as a contextual moderator, this framework captures the interaction between welfare-state design and service delivery in shaping fertility policy compliance. In this sense, the conceptual framework explicitly maps institutional variables onto key components of the Theory of Planned Behavior, treating fertility intentions as compliance-oriented behavioral intentions shaped by attitudes toward childbearing and perceived behavioral control under policy constraints.

**Figure 2:**
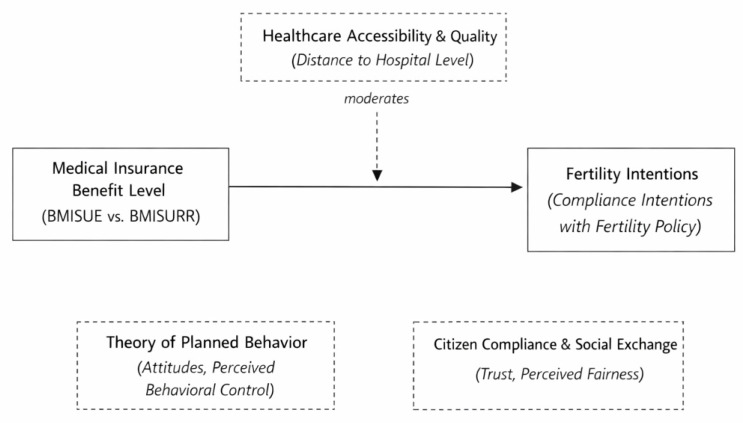
A Theoretical Framework of Healthcare Benefits and Compliance Willingness toward Fertility Policy.

At the construct level, the framework posits that (1) perceived financial protection and welfare support shape attitudinal evaluations of childbearing, and (2) perceived behavioral control over pregnancy, delivery, and childcare shapes fertility-related behavioral intentions. At the measurement level, insurance type (BMISUE vs. BMISURR) serves as a structural proxy for differential welfare generosity, while the tier of the nearest hospital proxies variation in healthcare accessibility. The framework further recognizes that several individual-level characteristics—including age, sex, marital status, parity, education, occupation, and income—are theoretically important confounders that are adjusted for in all regression models. These confounders are not depicted in the core causal pathway but are included in Figure 2 as a shaded “adjustment set” to clarify the analytical structure.

## 3. Materials and Methods

### 3.1. Data Source

**Figure 3:**
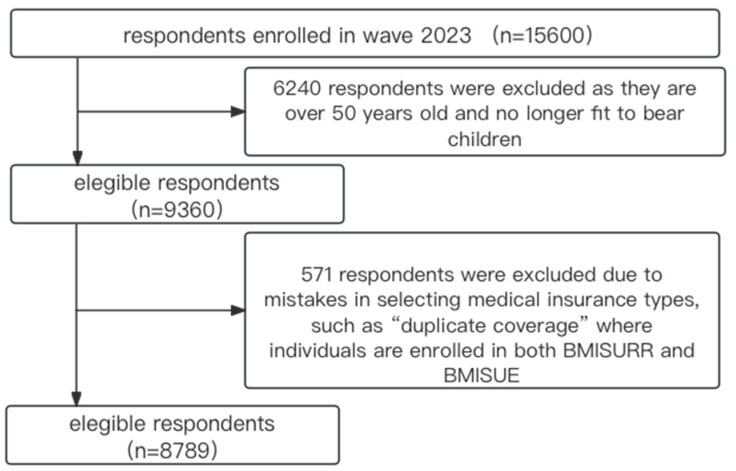
The flowchart of sample selection.

This study is a secondary analysis of data collected through an existing population-based survey. The original survey was designed to assess health life satisfaction among urban residents in the Yangtze River Delta and was not specifically designed to examine fertility intentions. The variables selected for the present analysis—insurance type, hospital proximity, and fertility intention—were available in the dataset and aligned with our analytical framework, though they were not purpose-designed to operationalize the theoretical constructs proposed here. The data used in this study were drawn from the “Health Life Satisfaction Survey of Yangtze River Delta (HLSSYRD)”, a two-phase survey conducted by Shanghai Jiao Tong University. The first phase (HLSSYRD I) was implemented in 2021 and focused primarily on residents’ health status, lifestyle, and general life satisfaction; it did not include items on fertility intentions, environmental perception, or social interaction. Therefore, that phase was not suitable for the present study. Instead, we used the second phase (HLSSYRD II), which was carried out in 2023. This survey employed a stratified, multistage, and probability-proportional-to-size sampling method, covering all urban residents in the Yangtze River Delta region. The questionnaire comprised ten sections covering basic demographic information, health insurance, choice of medical institutions, healthy environment, healthy society, and—importantly—fertility intentions and related psychosocial factors. A total of 18,638 questionnaires were distributed, and 15,600 valid responses were collected, giving a response rate of 84%. For the purposes of this study, we cleaned the data as follows (see Figure 3): we retained only respondents covered by either BMISUE or BMISURR, removed cases with duplicate enrolment, and excluded those who answered “don’t know” or “refuse to answer” or had missing values on key variables (e.g., fertility intention, income, and nearest hospital tier). After these exclusions, the final analytical sample consisted of 8789 participants.

The age cutoff of 50 years was applied based on China’s official reproductive age definition and is consistent with prior fertility intention studies in the Chinese context. We acknowledge that this criterion introduces a form of sample truncation; however, as the study concerns fertility intentions, including respondents who are biologically or socially unlikely to bear children would introduce noise rather than analytical value. Respondents with missing values on key variables (medical insurance type, nearest hospital, and the outcome measure) were excluded listwise. To assess whether this exclusion introduced systematic selection bias, we compared the characteristics of excluded and included respondents and found.

### 3.2. Variable Measurement

Since citizens’ fertility intentions to some extent reflect their fertility decisions [78] and consistent with the Theory of Planned Behavior, medical insurance benefit level is used as an institutional proxy for attitudes toward fertility, while proximity to different levels of medical institutions captures perceived behavioral control over childbearing. The dependent variable is individuals’ fertility intentions, which we interpret as a proxy for compliance-oriented behavioral intentions in the context of China’s pronatalist fertility policy. Fertility intentions were measured using the survey item: “Do you have plans or aspirations to have a child or additional children within the next few years?” Responses were dichotomized as 1 (Yes) and 0 (No). We acknowledge that stated fertility intentions may not fully capture policy compliance, as they reflect a broader set of reproductive preferences; however, they represent the closest available behavioral indicator in the current dataset.

The core explanatory variable of this study was the types of medical insurance. BMISUE targets formally employed urban workers and is financed through mandatory payroll contributions from both employers and employees. Coverage under BMISUE is individually based (tied to employment status rather than household registration), and it provides a personal medical savings account alongside a pooled insurance fund. The average inpatient reimbursement rate under BMISUE is approximately 80%. BMISUE offers comprehensive maternity benefits, including reimbursement of delivery costs, prenatal examinations, and postnatal care, as well as paid maternity leave entitlements linked to formal employment contracts. BMISURR, by contrast, covers non-working urban residents, rural residents, students, and the informally employed. It is financed primarily through individual voluntary contributions and government subsidies, with no employer component. BMISURR does not include a personal savings account, and its average inpatient reimbursement rate is approximately 70%, with lower ceilings and more limited maternity-related coverage. In this study, “insurance generosity” therefore refers specifically to this composite of reimbursement rates, maternity benefit scope, and overall benefit package comprehensiveness, not to any single dimension in isolation. The social medical insurance variable was coded as a binary indicator: BMISUE = 1, BMISURR = 0. In China’s current social health insurance system, BMISUE and BMISURR are intended to be mutually exclusive, as enrollment in BMISUE is linked to formal employment status. However, a subset of respondents (*n* = 571, approximately 6.1% of eligible respondents) reported dual coverage under both schemes. These individuals were excluded from the analytical sample because their dual enrollment likely reflects data entry errors or transitional coverage periods, and their benefit level cannot be unambiguously classified. This exclusion is illustrated in Figure 3. The final analytical sample therefore contains respondents exclusively enrolled in either BMISUE (*n* = 3937) or BMISURR (*n* = 4852).

Proximity to the nearest medical institution was assessed through the survey question: “What is the nearest medical institution to your residence?” Respondents selected from a predefined list of four categories: (1) tertiary hospitals, (2) secondary hospitals, (3) community health centres or township health centres, and (4) private clinics. For analytical purposes, community health centres, township health centres, and private clinics were grouped together as “primary-level institutions,” consistent with China’s three-tier hospital classification system [74]. This self-reported measure reflects respondents’ perception of their nearest facility rather than an objective geographic distance measurement, which represents a limitation discussed in Section 7. Furthermore, it should be noted that hospital tier is not a direct measure of reproductive or childcare service access. It captures neither antenatal care availability, postnatal support, delivery costs, nor waiting times. More importantly, hospital tier is likely to be spatially correlated with broader socioeconomic and urban development conditions: tertiary hospitals are predominantly located in highly developed urban districts characterized by higher housing costs, more intense labor-market competition, and greater socioeconomic inequality. The observed association between hospital tier and fertility intentions may therefore partly reflect these contextual factors rather than healthcare accessibility per se. This should be kept in mind when interpreting interaction effects involving hospital tier. Additionally, while all respondents are urban residents, the sample spans multiple cities within the Yangtze River Delta; city-level fixed effects were not included in the main models due to sample size constraints in subgroup analyses, though this represents a potential source of unobserved heterogeneity. In addition, we tried to control as much as possible for other relevant variables that may affect citizens’ fertility intentions, as shown in Table 1.

It should be acknowledged that the current dataset does not include direct survey items measuring individual-level psychological constructs such as attitudes toward childbearing, subjective norms, or perceived behavioral control in the sense originally proposed by Ajzen (1991) [65]. However, consistent with the institutionalized application of TPB in this study, medical insurance type serves as a structural proxy for attitudinal disposition toward fertility (reflecting perceived financial security and welfare support), while proximity to higher-tier hospitals serves as a structural proxy for perceived behavioral control (reflecting ease of accessing maternal and child health services). Future studies employing purpose-designed instruments could directly measure these psychological constructs to strengthen theoretical validity.

### 3.3. Analytical Plan

All analyses were conducted using Stata 15.0. The analytical strategy proceeded in three stages. First, descriptive statistics were used to characterize the sample’s sociodemographic profile, insurance enrollment, and hospital proximity (Table 1).

Second, binary logistic regression was employed as the primary analytical model, given the dichotomous nature of the outcome variable (fertility intention: yes/no). The main model (Model 1) regressed fertility intention on medical insurance type (BMISUE vs. BMISURR), adjusting for age, sex, marital status, parity, education, occupation, household income, self-rated physical health, and self-rated mental health. To examine whether the association between insurance type and fertility intention varied by healthcare accessibility, Model 2 introduced interaction terms between medical insurance type and the level of the nearest hospital. All logistic regression models were estimated using robust standard errors to account for potential heteroskedasticity. Results are presented as odds ratios (ORs) with 95% confidence intervals.

As a robustness check, probit regression models (Models 3 and 4) were estimated with identical specifications. Although logistic and probit models rely on different link functions (logistic vs. standard normal), they typically yield substantively consistent results for binary outcomes. Reporting both models allows assessment of whether findings are sensitive to the distributional assumption. For the probit models, we present exponentiated probit coefficients (exp(β)), which are reported solely to facilitate directional comparison with the logistic odds ratios; they do not constitute odds ratios and should not be interpreted as such. Readers primarily interested in magnitude should refer to the logistic regression results in Models 1 and 2. Average marginal effects for the probit models are available from the authors upon request and yield substantively identical conclusions regarding the direction and significance of the associations.

Third, heterogeneity analyses were conducted by stratifying the sample by (a) gender (female vs. male) and (b) number of existing children. These stratified analyses were exploratory in nature and were motivated by prior theoretical reasoning: insurance benefits may more strongly influence fertility intentions among women, given their direct exposure to maternity-related healthcare costs; and the parity composition of the sample (predominantly one-child families) suggests that the decision to have a second child may be particularly sensitive to institutional conditions. All subgroup analyses used the same model specifications as the main analysis.

The primary research objective is explanatory: to assess whether insurance benefit levels are associated with fertility intentions and whether this association is moderated by healthcare accessibility. The heterogeneity analyses by gender and parity are secondary and exploratory in nature; they were motivated by theoretical reasoning rather than pre-registered hypotheses.

## 4. Results

### 4.1. Descriptive Results

Table 1 presents the status of fertility intentions, medical insurance participation and the control variables of the study samples. With regard to fertility intentions, the majority of respondents (81.58%) reported having no desire for childbearing, while a smaller proportion (18.42%) expressed fertility intentions. Concerning medical insurance participation, 55.21% of the sample was enrolled in BMISURR, and 44.79% were covered by BMISUE. A substantial proportion (74.13%) reported that the closest hospital to their home was the primary hospital; then 12.93% and 12.95% of interviewees reported the secondary hospital and tertiary hospital, respectively.

In terms of control variables, the mean age of the respondents was 32.63 years (SD = 6.64), with a relatively balanced gender distribution (51.12% female, 48.88% male). The average family size was 3.32 members (SD = 0.86), and the majority (83.05%) were married. Regarding parity, 25.77% had never given birth, 60.26% had one child, 13.73% had two children, and only 0.24% had three or more children. The sample was relatively well-educated, with 50.85% holding a four-year college degree or higher. Most respondents were primarily in self-employment (56.79%) and public sector occupations (18.24%). Monthly household income was concentrated in the ranges of 10,001–20,000 CNY (61.38%) and 20,001–40,000 CNY (23.39%). Furthermore, 86.20% rated their health as good, and 76.41% reported good mental health.

Age was included as a continuous variable, given its well-documented nonlinear relationship with fertility intentions; older respondents are less likely to intend further childbearing as they approach the end of their reproductive years. Monthly household income was treated as an ordinal variable (four categories) due to its original survey coding. Linearity was assessed by comparing models with linear versus categorical income specifications; the categorical approach was retained as it better captured the non-proportional income gradient observed in preliminary analyses. Parity (Birth Children) was treated as an ordinal variable (0 = never, 1 = one child, 2 = two children, 3 = three or more), reflecting its role as both a social norm indicator and a demographic constraint on further fertility. All categorical variables are included as dummy-coded indicators with reference categories as reported in Table 1.

Table 2 presents the baseline characteristics of the analytic sample stratified by insurance type. Statistically significant differences were observed between BMISURR enrollees (*n* = 4852) and BMISUE enrollees (*n* = 3937) across all variables examined (all *p* < 0.001). BMISUE enrollees were older (median age: 34 years [IQR: 30–39] vs. 30 years [IQR: 26–35]) and more likely to be married (89.1% vs. 78.1%). Educational attainment was substantially higher in the BMISUE group, with 61.2% holding a college degree or above, compared with 42.4% among BMISURR enrollees. The occupational profiles of the two groups diverged markedly: BMISUE enrollees were disproportionately concentrated in formal-sector employment (Occupation Type 2: 31.9% vs. 7.2%), while BMISURR enrollees predominantly held informal or agricultural occupations (Occupation Type 3: 65.8% vs. 45.7%). Consistent with these patterns, BMISUE enrollees reported considerably higher monthly household income, with 35.7% falling in the two highest income categories (Categories 4–5: 31.5% + 4.2%) compared with 18.2% of BMISURR enrollees (16.8% + 1.4%). With respect to healthcare access, BMISUE enrollees were more likely to have a secondary- or tertiary-level hospital as their nearest facility (Level 2: 16.8% vs. 9.8%; Level 3: 19.0% vs. 8.0%), indicating substantially greater proximity to higher-quality healthcare services. Despite being older, BMISUE enrollees reported poorer self-rated physical health (good health: 79.2% vs. 91.8%) and mental health (good mental health: 73.8% vs. 78.6%), a pattern likely attributable to greater morbidity awareness and healthcare utilisation among formally employed, older individuals. The prevalence of positive fertility intentions was higher among BMISUE enrollees (21.3% vs. 16.1%). Taken together, these baseline differences confirm systematic socioeconomic and healthcare access disparities between the two insurance groups, underscoring the necessity of comprehensive covariate adjustment in subsequent analyses.

### 4.2. Regression Results

#### 4.2.1. Main Effects of Medical Insurance and Hospital Proximity

Using maximum likelihood estimation, we examined the association between medical insurance type, proximity to hospital tier, and individual fertility intentions. Results are presented in Table 3. In the column model (1), individuals enrolled in the BMISUE exhibited significantly higher odds of having fertility intentions compared to those enrolled in the BMISURR (OR = 1.2154, 95% CI = 1.0626–1.3901, *p* < 0.001). Several socio-demographic characteristics were also associated with fertility intentions. Older age was negatively associated with fertility intentions (OR = 0.9189, 95% CI = 0.9068–0.9312, *p* < 0.001), while being male (OR = 1.2015, 95% CI = 1.0670–1.3529, *p* < 0.001) and being married (OR = 9.1754, 95% CI = 7.0331–11.9702, *p* < 0.001) were positively associated with higher fertility intentions. Additionally, a higher number of existing children was associated with lower odds of fertility intentions (OR = 0.3200, 95% CI = 0.2667–0.3839, *p* < 0.001). Regarding educational attainment, individuals with high school/vocational education (OR = 0.4054, 95% CI = 0.2085–0.7882, *p* < 0.001) had lower fertility intentions compared to those with middle school education or below. Furthermore, self-employed individuals (OR = 0.7543, 95% CI = 0.6064–0.9381, *p* < 0.05) and students (OR = 0.3573, 95% CI = 0.2185–0.5842, *p* < 0.001) had significantly lower fertility intentions compared to workers and farmers. In contrast, individuals with other occupations displayed higher fertility intentions (OR = 1.5199, 95% CI = 1.1779–1.9613, *p* < 0.001) compared to workers and farmers. Higher monthly household income was associated with higher fertility intentions (OR = 1.6277, 95% CI = 1.4786–1.7919, *p* < 0.001). Individuals with better self-rated health (OR = 0.5618, 95% CI = 0.4728–0.6676, *p* < 0.001) and better self-rated mental health (OR = 0.7862, 95% CI = 0.6772–0.9127, *p* < 0.001) had lower fertility intentions. The analyses were based on 8789 observations.

#### 4.2.2. Analysis of the Moderating Role of Healthcare Accessibility

In the column model (2), which included interaction terms between medical insurance and the level of the nearest hospital, individuals enrolled in BMISUE and living near secondary hospitals exhibited significantly higher odds of fertility intentions (OR = 1.6727, 95% CI = 1.1273–2.4821, *p* < 0.05) compared to those enrolled in BMISURR and living near primary hospitals. However, no significant difference was observed for individuals enrolled in BMISUE and living near tertiary hospitals compared to the reference group. The directions and significance levels of the other control variables remained similar to those in the column model (2).

#### 4.2.3. Robustness Check

The results from the probit models were generally consistent with those from the column model (3) and the column model (4). Individuals enrolled in BMISUE exhibited higher fertility intentions compared to those enrolled in BMISURR (model 3: OR = 1.1133, 95% CI = 1.0326–1.2004, *p* < 0.001; model 4: OR = 1.0069, 95% CI = 0.9207–1.1013, *p* > 0.05). Additionally, those enrolled in BMISUE and living near secondary hospitals had significantly higher fertility intentions (model 4: OR = 1.3937, 95% CI = 1.1244–1.7274, *p* < 0.001) compared to the reference group.

### 4.3. Heterogeneity Analysis

#### 4.3.1. Gender-Based Heterogeneity Analysis

Table 4 delineated the relationship between medical insurance, the nearest hospital and fertility intentions of individuals, segmented by gender. Columns (5) and (7) show that the association between medical insurance and fertility intentions, there was a notable gender difference. For females (model 5), enrollment in BMISUE was associated with significantly higher odds of fertility intentions compared to BMISURR (OR = 1.2749, 95% CI = 1.0525–1.5442, *p* < 0.05). However, for males (model 7), there was no significant difference in fertility intentions between those enrolled in BMISUE and BMISURR.

When considering the moderating effect of the nearest hospital level, the interaction patterns differed between males and females. For females (column 6), those enrolled in BMISUE and living near tertiary hospitals exhibited significantly higher fertility intentions compared to those enrolled in BMISURR and living near primary hospitals (OR = 2.8625, 95% CI = 1.6506–4.9641, *p* < 0.001). In contrast, for males (column 8), those enrolled in BMISUE and living near secondary hospitals had higher fertility intentions than the reference group (OR = 1.9610, 95% CI = 1.0471–3.6727, *p* < 0.05), while those living near tertiary hospitals had lower fertility intentions (OR = 0.5935, 95% CI = 0.3712–0.9489, *p* < 0.05).

The negative association between proximity to tertiary hospitals and fertility intentions among males may reflect the broader socioeconomic contexts in which such hospitals are embedded rather than the effect of healthcare accessibility per se. In China, tertiary hospitals are predominantly located in highly urbanized areas characterized by intense labor-market competition, long working hours, and high opportunity costs of family formation. Prior studies consistently show that men’s fertility intentions are particularly sensitive to employment pressure, economic insecurity, and work–family conflict, especially in urban settings [6,34]. Moreover, gendered reproductive roles suggest that men tend to evaluate fertility decisions primarily through the lens of financial responsibility and breadwinner expectations rather than healthcare availability [79]. As a result, living near tertiary hospitals—often associated with higher living costs and intensified work demands—may dampen men’s fertility intentions despite improved medical resources. This finding is consistent with prior evidence that fertility responses to institutional conditions are highly gendered and context-dependent rather than uniformly positive [71,80].

#### 4.3.2. Heterogeneity Analysis by Number of Existing Children

The association between medical insurance and fertility intentions varied based on individuals’ existing number of children. For those who never had children (column 9), there was no significant difference in fertility intentions between those enrolled in BMISUE and BMISURR. However, for individuals with one child (column 11), enrollment in BMISUE was associated with significantly higher odds of intending to have another child compared to those enrolled in BMISURR (OR = 1.3987, 95% CI = 1.1756–1.6643, *p* < 0.001). In contrast, among those with two children (column 13), individuals enrolled in BMISUE had lower fertility intentions than those in BMISURR, although the difference was not statistically significant.

Furthermore, for those who never had children (column 10), there was no significant interaction between medical insurance type and hospital level. Among individuals with one child (column 12), the interaction effects were also non-significant. However, for those with two children (column 14), individuals enrolled in BMISUE and living near secondary hospitals exhibited significantly higher fertility intentions than the reference group of those enrolled in BMISURR and living near primary hospitals (OR = 5.1249, 95% CI = 1.3843–18.9736, *p* < 0.05). The interaction between BMISUE and living near tertiary hospitals was not significant for those with two children (Table 5).

## 5. Discussion

Our study findings support previous research highlighting significant associations between medical insurance benefits and individuals’ fertility intentions. We found that individuals enrolled in medical insurance schemes with higher benefit levels, such as the BMISUE scheme, showed a greater willingness to have children compared to those in schemes with lower benefit levels, like the BMISURR scheme [34,37,81]. This finding is consistent with the idea that enhanced healthcare access and financial security may be associated with reduced concerns related to childbearing and encourage individuals to consider expanding their families [35,82]. The variations in fertility intentions among different medical insurance schemes can be attributed to how citizens perceive the fairness and equity of the healthcare system. Those with more comprehensive medical insurance benefits may view the system as more equitable and supportive, which in turn builds trust and a greater willingness to have children [21,22,83]. On the other hand, individuals with limited medical insurance coverage may see the system as unfair, leading to lower trust and reduced fertility intentions [29]. Our study adds to the existing literature exploring the relationship between healthcare factors and fertility decisions [35,37,82]. By specifically focusing on individuals’ fertility intentions, our research highlights the importance of medical insurance benefits and healthcare accessibility in influencing reproductive choices, an area that has received limited attention in previous studies. The study’s results hold significant policy implications for governments aiming to encourage desired fertility rates. Enhancing medical insurance benefits can help alleviate financial and health-related worries linked to childbearing and childrearing. Additionally, this can cultivate a perception of equity and confidence in the healthcare system, potentially boosting individuals’ inclination to start a family [37,83].

Furthermore, our study’s findings contribute to the expanding literature on the interplay among healthcare service quality, accessibility, and individuals’ compliance with government fertility policies. Consistent with prior research, our findings suggest that perceived healthcare accessibility is positively associated with fertility intentions. The moderation analysis revealed that the positive association between insurance benefit levels and fertility intentions was more pronounced among individuals residing near secondary hospitals, suggesting that the practical credibility of welfare entitlements depends on the local service delivery context. These findings do not establish a causal pathway, but they point to the importance of jointly considering welfare design and service accessibility in understanding reproductive decision-making [83,84]. The moderation analysis further revealed that the positive association between medical insurance benefit levels and compliance with fertility policies was more pronounced when individuals had access to secondary hospitals in close proximity to their homes. This finding suggests that the perceived quality and accessibility of healthcare services may strengthen the association between financial protection and fertility intentions [85,86,87]. When individuals perceive healthcare services as high-quality and easily accessible, they may view government fertility policies as more feasible and less burdensome [88]. Access to reliable and affordable healthcare may be associated with reduced concerns related to the potential health risks and financial costs associated with childbearing and childrearing, thereby increasing individuals’ willingness to comply with policies that encourage or discourage fertility [35,89]. Moreover, positive perceptions of healthcare service quality and accessibility can foster trust in the government’s ability to provide adequate healthcare support for families. When individuals trust that the healthcare system will meet their needs during and after childbearing, they may be more inclined to adhere to fertility policies, as they perceive the government as capable of delivering on its promises and commitments [90]. Positive experiences with healthcare providers, efficient service delivery, and supportive social networks can foster favorable perceptions, while negative experiences, long waiting times, and lack of social support can lead to unfavorable perceptions and reduced compliance with fertility policies.

The negative association between better self-rated health and fertility intentions, while seemingly counterintuitive, is not inconsistent with the existing literature. Previous studies report mixed evidence: some find that good physical and mental health facilitates fertility intentions by reducing perceived biological risks [78,80], whereas others document a negative or context-dependent relationship in low-fertility, highly developed societies [34,91]. In such contexts, good self-rated health may signal stronger labor-market attachment, higher opportunity costs, and preferences for maintaining lifestyle quality, thereby discouraging childbearing [82]. Conversely, individuals with poorer self-rated health may place greater value on family formation as a source of emotional support and life meaning [36]. These findings suggest that the relationship between health and fertility intentions is multidimensional and highly context-dependent rather than uniformly positive.

The Chinese cultural context surrounding pregnancy and childbirth provides additional insights into this discovery. In traditional Chinese families, it is common for young couples to seek support from their parents during the postpartum period, known as ‘zuo yuezi’ or ‘sitting the month’ [92]. New mothers typically receive help from their mothers or mothers-in-law in caring for the newborn and recovering from childbirth during this time. Therefore, when considering fertility intentions, young couples may take into account the availability of healthcare services not only for themselves but also for their parents, who may play a vital role in providing postpartum support. If high-quality secondary hospitals are conveniently located near the couple’s residence, it may ease concerns about accessing adequate healthcare for both themselves and their parents during the demanding postpartum period. This perception of readily available and reliable healthcare resources can lead to a greater willingness to have children, especially for those with higher medical insurance benefits (BMISUE) who may have better financial protection and access to superior healthcare services [93]. Our findings add to the expanding body of literature examining the influence of healthcare-related factors on individuals’ fertility decisions, particularly in developing countries with diverse cultural and healthcare environments. By taking into consideration the unique cultural context of postpartum care in China and the perceived quality and accessibility of secondary hospitals, our study offers insights into the complex interplay between healthcare factors and fertility intentions.

## 6. Conclusions

Our findings indicate a significant positive association between medical insurance benefit levels and individuals’ fertility intentions within China’s pronatalist policy context. Specifically, individuals covered by BMISUE, which offers higher benefit levels, reported higher fertility intentions than those covered by BMISURR. These associations are consistent with the view that institutional welfare arrangements may shape individuals’ reproductive decision-making, though the cross-sectional design precludes causal interpretation. These findings suggest that fragmented welfare entitlements and unequal healthcare accessibility may constrain the effectiveness of pronatalist policy incentives. Policies that narrow the benefit gap between BMISUE and BMISURR—for example, by improving maternity reimbursement rates and extending formal maternity entitlements to non-employed residents—may be associated with increased fertility intentions, particularly among groups currently underserved by the welfare system. However, given the cross-sectional and observational nature of this study, we cannot establish that such policy changes will causally increase birth rates. The associations documented here should be interpreted as indicative of potentially important institutional pathways, warranting evaluation through longitudinal or quasi-experimental research designs. Our research findings indicate that access to quality medical resources can positively moderate the relationship between medical insurance benefits and citizens’ willingness to comply with fertility policies. This moderating effect is particularly pronounced for secondary healthcare institutions. Based on these findings, we propose the following policy recommendations to enhance primary healthcare capacity, provide quality medical resources, and consequently increase fertility intentions among the Chinese population.

By examining the moderating influence of healthcare accessibility, our research sheds light on the circumstances in which medical insurance benefits could have a more significant impact on individuals’ willingness to adhere to fertility policies. These findings hold significant policy implications for governments aiming to encourage fertility rates and boost compliance with fertility policies. For international readers, the broader policy implication is that fragmented social insurance systems and spatially unequal healthcare infrastructure may undermine the intended effects of pronatalist policies. These structural conditions—rather than individual-level preferences alone—may help explain persistent low fertility in rapidly developing economies.

## 7. Limitations

This study has several inevitable limitations that should be acknowledged. First, the analysis is based on cross-sectional survey data, which constrains the ability to draw strong causal inferences regarding the relationships among medical insurance type, healthcare accessibility, and fertility intentions. Although the empirical results reveal systematic and theoretically consistent associations, these relationships should be interpreted as correlational rather than causal. In particular, the cross-sectional design does not allow for the examination of temporal ordering or dynamic changes in fertility intentions over time. Moreover, unobserved individual characteristics—such as underlying health status, long-term fertility preferences, or unmeasured family considerations—as well as broader contextual factors may partially influence both insurance participation and fertility intentions, and cannot be fully accounted for within this framework.

Second, this study focuses on citizens’ fertility intentions within China’s Yangtze River Delta region, a highly developed area with relatively abundant healthcare resources and comparatively generous welfare provision. While this regional focus allows for a more context-sensitive examination of institutional effects, it may also limit the generalizability of the findings to less-developed regions within China or to other national contexts. Although East Asian societies share certain cultural and institutional features related to family formation and state involvement, notable cross-national and subnational differences remain in healthcare systems, welfare regimes, and fertility policy designs. Future research could extend the analysis to other regions of China or adopt a cross-national comparative approach to assess whether the observed relationships hold under different institutional and cultural conditions.

Third, healthcare accessibility and quality are proxied by the hierarchical level of the nearest medical institution, which represents a simplified measure of a multifaceted concept. While hospital tier captures important differences in service capacity, specialization, and perceived quality within China’s healthcare system, it does not account for other dimensions of accessibility, such as waiting times, service costs, provider–patient ratios, or subjective evaluations of care. Future research could employ more granular indicators, including travel time, healthcare utilization experiences, or patient satisfaction measures, to more comprehensively assess healthcare accessibility. Specifically, the nearest hospital tier variable may capture urbanization level, local housing costs, and labor-market pressures—factors that have been independently associated with fertility intentions, particularly among men. Future research should employ geographically linked data on actual reproductive health service availability, travel time, and healthcare utilization to more precisely isolate the healthcare accessibility pathway from broader urban socioeconomic effects.

Despite these limitations, this study provides meaningful insights into the institutional foundations of fertility policy compliance by highlighting the role of medical insurance differentiation and healthcare accessibility. Addressing these limitations through longitudinal data, quasi-experimental designs, or broader geographic coverage would further strengthen causal claims and enhance the external validity of future research.

## Figures and Tables

**Table 1:** Definition and descriptive results of variables.

Variables	Definitions	*n*/Mean	Percent/SE
Fertility Intentions	No = 0	7170	81.58%
	Yes = 1	1619	18.42%
Medical Insurance	BMISURR = 0	4852	55.21%
	BMISUE = 1	3937	44.79%
The Nearest Hospital	Primary Hospital = 1	6515	74.13%
	Secondary Hospital = 2	1136	12.93%
	Tertiary Hospital = 3	1138	12.95%
Age	Continuous Variable	32.63	6.64
Gender	Female = 0	4493	51.12%
	Male = 1	4296	48.88%
Family Population	Continuous Variable	3.32	0.86
Marital status	Unmarried = 0	1490	16.95%
	Married = 1	7299	83.05%
Birth Children	Never = 1	2265	25.77%
	Have One Child = 2	5296	60.26%
	Have Two Children = 3	1207	13.73%
	Have Three Children and above = 4	21	0.24%
Education Level	Middle school and below = 1	84	0.96%
	High school/Vocational school = 2	1098	12.49%
	Three-year college = 3	3138	35.70%
	Four-year college and above = 4	4469	50.85%
Occupation	Workers and Farmers = 1	890	10.13%
	Public Sector Employees = 2	1603	18.24%
	Self-Employed Individuals = 3	4991	56.79%
	Students = 4	363	4.12%
	Other Occupations = 5	943	10.73%
Monthly Household Income	0–10,000 CNY = 1	1106	12.58%
	10,001–20,000 CNY = 2	5395	61.38%
	20,001–40,000 CNY = 3	2056	23.39%
	40,001 CNY and above = 4	232	2.64%
Self-rated health	Poor = 0	1213	13.80%
	Good = 1	7576	86.20%
Self-rated Mental health	Poor = 0	2073	23.59%
	Good = 1	6716	76.41%
*n*		8789	100%

Note: For categorical variables, frequencies (*n*) and valid percentages (%) are reported. For continuous variables, means and standard deviations (SD) are reported. Percentages within each variable may not sum to 100% due to rounding.

**Table 2:** Baseline characteristics of participants by medical insurance type.

Variable	BMISURR (*n* = 4852)	BMISUE (*n* = 3937)	*p*-Value
Age median	30 (26–35)	34 (30–39)	<0.001
Family size median	3 (3–4)	3 (3–4)	<0.001
Number of children median	2 (1–2)	2 (2–2)	<0.001
Gender Male (%)	2449 (50.5%)	1847 (46.9%)	<0.001
Gender Female (%)	2403 (49.5%)	2090 (53.1%)	<0.001
Marital status Unmarried/divorced/widowed (%)	1062 (21.9%)	428 (10.9%)	<0.001
Marital status Married/cohabiting (%)	3790 (78.1%)	3509 (89.1%)	<0.001
Education Primary or below (%)	37 (0.8%)	47 (1.2%)	<0.001
Education Junior high (%)	633 (13.0%)	465 (11.8%)	<0.001
Education Senior high (%)	2124 (43.8%)	1014 (25.8%)	<0.001
Education College and above (%)	2058 (42.4%)	2411 (61.2%)	<0.001
Occupation Government/public institution (%)	374 (7.7%)	516 (13.1%)	<0.001
Occupation State-owned enterprise (%)	349 (7.2%)	1254 (31.9%)	<0.001
Occupation Private/self-employed (%)	3193 (65.8%)	1798 (45.7%)	<0.001
Occupation Unemployed/retired (%)	342 (7.0%)	20 (0.5%)	<0.001
Occupation Other (%)	594 (12.2%)	349 (8.9%)	<0.001
Household income <5000 CNY (%)	751 (15.5%)	355 (9.0%)	<0.001
Household income 5000–10,000 CNY (%)	3218 (66.3%)	2177 (55.3%)	<0.001
Household income 10,000–20,000 CNY (%)	816 (16.8%)	1240 (31.5%)	<0.001
Household income >20,000 CNY (%)	67 (1.4%)	165 (4.2%)	<0.001
Nearest hospital Primary (%)	3987 (82.2%)	2528 (64.2%)	<0.001
Nearest hospital Secondary (%)	476 (9.8%)	660 (16.8%)	<0.001
Nearest hospital Tertiary (%)	389 (8.0%)	749 (19.0%)	<0.001
Self-rated health Good (%)	4456 (91.8%)	3120 (79.2%)	<0.001
Mental health Good (%)	3812 (78.6%)	2904 (73.8%)	<0.001

**Table 3:** The regression results for the association between medical insurance, the nearest hospital and fertility intentions.

	(1)	(2)	(3)	(4)
	Logit	Logit	Probit	Probit
VARIABLES	Fertility Intentions	Fertility Intentions	Fertility Intentions	Fertility Intentions
Ref: Medical insurance (BMISURR)				
BMISUE	1.2154 ***	1.0306	1.1133 ***	1.0069
	[1.0626- 1.3901]	[0.8772–1.2109]	[1.0326–1.2004]	[0.9207–1.1013]
Ref: The Nearest Hospital (Primary Hospital)				
2. The Nearest Hospital		0.8016		0.8721
(Secondary Hospital)		[0.5838–1.1007]		[0.7370–1.0320]
3. The Nearest Hospital		1.4182 **		1.1990 **
(Tertiary Hospital)		[1.0575–1.9021]		[1.0160–1.4151]
Ref: BMISURR # The Nearest Hospital				
BMISUE # Secondary Hospital		1.6727 **		1.3937 ***
		[1.1273–2.4821]		[1.1244–1.7274]
BMISUE # Tertiary Hospital		1.2939		1.1805
		[0.9084–1.8430]		[0.9655–1.4434]
Age	0.9189 ***	0.9190 ***	0.9528 ***	0.9529 ***
	[0.9068–0.9312]	[0.9068–0.9314]	[0.9459–0.9596]	[0.9460–0.9598]
Ref: Female				
Male	1.2015 ***	1.1919 ***	1.1035 ***	1.1005 ***
	[1.0670–1.3529]	[1.0580–1.3428]	[1.0323–1.1797]	[1.0293–1.1767]
Family Population	1.0776	1.0894 *	1.0333	1.0401
	[0.9825–1.1819]	[0.9919–1.1965]	[0.9805–1.0890]	[0.9864–1.0967]
Ref: Unmarried				
Married	9.1754 ***	9.8056 ***	3.3140 ***	3.4215 ***
	[7.0331–11.9702]	[7.4705–12.8706]	[2.8500–3.8536]	[2.9354–3.9882]
Birth Children	0.3200 ***	0.3134 ***	0.5469 ***	0.5400 ***
	[0.2667–0.3839]	[0.2610–0.3762]	[0.4938–0.6057]	[0.4875–0.5982]
Ref: Middle school and below				
High school/Vocational school	0.4054 ***	0.3741 ***	0.5974 ***	0.5745 ***
	[0.2085–0.7882]	[0.1940–0.7212]	[0.4100–0.8705]	[0.3961–0.8332]
Three-year college	0.7865	0.7097	0.8448	0.8027
	[0.4182–1.4792]	[0.3807–1.3231]	[0.5887–1.2121]	[0.5620–1.1464]
Four-year college and above	0.9101	0.8027	0.9018	0.8435
	[0.4851–1.7073]	[0.4318–1.4923]	[0.6293–1.2922]	[0.5914–1.2031]
Ref: Workers and Farmers				
Public Sector Employees	1.1883	1.1874	1.1136	1.1136
	[0.9327–1.5139]	[0.9311–1.5143]	[0.9728–1.2747]	[0.9724–1.2752]
Self-Employed Individuals	0.7543 **	0.7539 **	0.8412 ***	0.8399 ***
	[0.6064–0.9381]	[0.6038–0.9412]	[0.7455–0.9491]	[0.7430–0.9495]
Students	0.3573 ***	0.3801 ***	0.5680 ***	0.5819 ***
	[0.2185–0.5842]	[0.2323–0.6218]	[0.4406–0.7322]	[0.4510–0.7506]
Other Occupations	1.5199 ***	1.5489 ***	1.2497 ***	1.2584 ***
	[1.1779–1.9613]	[1.1970–2.0042]	[1.0826–1.4427]	[1.0885–1.4547]
Monthly Household Income	1.6277 ***	1.5778 ***	1.3167 ***	1.2976 ***
	[1.4786–1.7919]	[1.4297–1.7412]	[1.2473–1.3898]	[1.2280–1.3711]
Self-rated Health	0.5618 ***	0.5738 ***	0.7128 ***	0.7211 ***
	[0.4728–0.6676]	[0.4823–0.6827]	[0.6458–0.7867]	[0.6528–0.7965]
Self-rated Mental Health	0.7862 ***	0.7788 ***	0.8789 ***	0.8761 ***
	[0.6772–0.9127]	[0.6703–0.9048]	[0.8081–0.9559]	[0.8053–0.9532]
Observations	8789	8789	8789	8789

Robust in brackets *** *p* < 0.01, ** *p* < 0.05, * *p* < 0.1. # indicates a moderating effect (interaction term).

**Table 4:** Heterogeneity Analysis by Gender.

	(5)	(6)	(7)	(8)
	Female	Female	Male	Male
VARIABLES	Fertility Intentions	Fertility Intentions	Fertility Intentions	Fertility Intentions
Ref: Medical insurance (BMISURR)				
BMISUE	1.2749 **	0.9870	1.1627	1.1182
	[1.0525–1.5442]	[0.7823–1.2454]	[0.9580–1.4112]	[0.8910–1.4033]
Ref: The Nearest Hospital (Primary Hospital)				
The Nearest Hospital		0.9808		0.6609
(Secondary Hospital)		[0.6446–1.4924]		[0.3919–1.1147]
The Nearest Hospital		0.8698		2.1979 ***
(Tertiary Hospital)		[0.5423–1.3951]		[1.5022–3.2157]
Ref: BMISURR # The Nearest Hospital				
BMISUE # Secondary Hospital		1.3198		1.9610 **
		[0.7772–2.2413]		[1.0471–3.6727]
BMISUE # Tertiary Hospital		2.8625 ***		0.5935 **
		[1.6506–4.9641]		[0.3712–0.9489]
Other variables	Yes			
Observations	4493	4493	4296	4296

Robust in brackets *** *p* < 0.01, ** *p* < 0.05. # indicates a moderating effect (interaction term).

**Table 5:** Heterogeneity Analysis by Number of Existing Children.

	(9)	(10)	(11)	(12)	(13)	(14)
	Never	Never	Have One Child	Have One Child	Have Two Children	Have Two Children
VARIABLES	Fertility Intentions	Fertility Intentions	Fertility Intentions	Fertility Intentions	Fertility Intentions	Fertility Intentions
Ref: Medical insurance (BMISURR)						
BMISUE	0.9780	0.8437	1.3987 ***	1.2577 **	0.7127	0.4889 **
	[0.7395–1.2935]	[0.5980–1.1904]	[1.1756–1.6643]	[1.0197–1.5512]	[0.4212–1.2059]	[0.2654–0.9003]
Ref: The Nearest Hospital (Primary Hospital)						
2. The Nearest Hospital		0.5852 *		1.0498	0.4081 *	0.4081 *
(Secondary Hospital)		[0.3330–1.0282]		[0.6864–1.6056]	[0.1646–1.0121]	[0.1646–1.0121]
The Nearest Hospital		1.0677		2.2011 ***	1.4776	1.4776
(Tertiary Hospital)		[0.6554–1.7393]		[1.4769–3.2804]	[0.4320–5.0540]	[0.4320–5.0540]
Ref: BMISURR # The Nearest Hospital						
BMISUE # Secondary Hospital		1.4258		1.4066		5.1249 **
		[0.6628–3.0672]		[0.8446–2.3426]		[1.3843–18.9736]
BMISUE # Tertiary Hospital		1.6302		0.8854		1.1381
		[0.8529–3.1159]		[0.5552–1.4118]		[0.2669–4.8528]
Other variables		Yes				
Observations	2265	2265	5294	5294	1179	1179

Robust in brackets *** *p* < 0.01, ** *p* < 0.05, * *p* < 0.1. # indicates a moderating effect (interaction term).

## Data Availability

The data presented in this study are available on request from the corresponding author due to privacy and ethical restrictions.

## Abbreviations

BMISUE	Basic Medical Insurance System for Urban Employees
BMISURR	Basic Medical Insurance System for Urban and Rural Residents
HLSSYRD	Health Life Satisfaction Survey of Yangtze River Delta
HLSSYRD II	Health Life Satisfaction Survey of Yangtze River Delta Phase II
NRCMS	New Rural Cooperative Medical Scheme
TFR	Total Fertility Rate
YRDC	Yangtze River Delta in China
